# The Effectiveness of Lurasidone Add-On for Residual Aggressive Behavior and Obsessive Symptoms in Antipsychotic-Treated Children and Adolescents with Tourette Syndrome: Preliminary Evidence from a Case Series

**DOI:** 10.3390/children8020121

**Published:** 2021-02-09

**Authors:** Marco Colizzi, Riccardo Bortoletto, Leonardo Zoccante

**Affiliations:** 1 Section of Psychiatry, Department of Neurosciences, Biomedicine and Movement Sciences, University of Verona, 37134 Verona, Italy; 2 Department of Psychosis Studies, Institute of Psychiatry, Psychology and Neuroscience, King’s College London, London SE5 8AF, UK; 3 Child and Adolescent Neuropsychiatry Unit, Maternal-Child Integrated Care Department, Integrated University Hospital of Verona, 37126 Verona, Italy; riccardo.bortoletto91@gmail.com (R.B.); leonardo.zoccante@aovr.veneto.it (L.Z.)

**Keywords:** treatment resistant Tourette disorder, psychopharmacological treatment, aripiprazole, risperidone, case report

## Abstract

Children and adolescents with Tourette syndrome may suffer from comorbid psychological and behavioral difficulties, primarily Attention-Deficit Hyperactivity Disorder-related manifestations including impulsive, aggressive, and disruptive behavior, and Obsessive-Compulsive Disorder-related disturbances. Often, such additional problems represent the major cause of disability, requiring their prioritization above the tic symptomatology. Here, we present six cases of children and adolescents with treatment-resistant Tourette syndrome aged 11–17 years, whose symptoms, especially the non-tic symptoms such as aggressive behavior and obsessive symptoms, failed to respond adequately to at least two different antipsychotics and, where deemed appropriate, to a combination with a medication with a different therapeutic indication or chemical class (e.g., antidepressant or anticonvulsant). Such symptomatic manifestations were significantly reduced by the time of the subsequent control visit planned 30 days later, by using lurasidone as an add-on therapy to risperidone or aripiprazole (all *p* ≤ 0.009). No significant neuromotor or metabolic side effects were reported in all cases in a follow-up period ranging from 4 months to 6 months, supporting the stability of the observed clinical improvement. While still investigational, the preliminary evidence presented here gives reason to hope that lurasidone could possibly be an effective option in Tourette syndrome, warranting further investigation of its potential benefits in neurodevelopmental conditions.

## 1. Introduction

Beyond pathognomonic motor and vocal tics, children and adolescents with Tourette syndrome (TS) suffer from comorbid psychological and behavioral difficulties, primarily Attention-Deficit Hyperactivity Disorder-related manifestations including impulsive, aggressive, and disruptive behavior, and Obsessive-Compulsive Disorder-related disturbances. Other symptoms less commonly reported are anxiety, mood swings, and personality alterations. Such additional problems often represent the major cause of disability, requiring their prioritization above the tic symptomatology [1].

TS treatment can be conceptualized in three phases. First, psychosocial interventions (e.g., psychoeducation and social support) should be offered to all TS individuals and their families. Second, depending on the symptom severity and related impairment, active interventions can be pursued, including behavioral and pharmacological therapies. Antipsychotics are the most commonly used medications, followed by alpha agonists and other compounds including anticonvulsants, muscle relaxant agents, cannabis-derived formulations, and immunoglobulins. Third, when TS is refractory to both behavioral and pharmacological treatments, individuals may be eligible for neuromodulation interventions. However, such interventions can be invasive and less feasible in common clinical settings, and there is uncertainty regarding the selection of patients and target of intervention [2].

Most of the evidence regarding the efficacy of antipsychotic drugs in TS comes from studies with haloperidol, pimozide, risperidone, and aripiprazole. More limited evidence also supports the use of ziprasidone, tiapride, and metoclopramide. While risperidone and aripiprazole are Food and Drug Administration (FDA)-approved medications for behavioral disturbances other than tics in children and adolescents, there is no solid evidence to establish superiority of one medication relative to another and side effects have been reported for all tested medications. Importantly, because of the phenotypic variability, no antipsychotic has proven effective for all patients with TS [3]. The last decade has seen the approval of new antipsychotic drugs, one of which is lurasidone. Unique to lurasidone is a potent antagonism at the serotonin 5-HT7 receptor, which, coupled with 5-HT1A partial agonism, could potentially be associated with additional cognitive-enhancing and antidepressant effects. In addition, lurasidone shows low affinity for the M1, H1, 5-HT2C, and α1 receptors, suggesting a low liability to cause peripheral and central anticholinergic side effects, somnolence, weight gain, and hypotension [4].

Evidence indicates that antipsychotic polypharmacy (APP), i.e., the co-prescription of more than one antipsychotic medication for an individual patient, is a relatively frequent and consistent practice in adults, with a prevalence of up to 50% in clinical settings devoted to the treatment of severe psychosis [5]. While current treatment guidelines state that antipsychotic monotherapy (APM) should be preferred to APP, based on high-quality studies of the *acute-phase treatment*, recent evidence from a 20-year follow-up study conducted among a nationwide cohort of 62,250 psychosis patients indicates that certain APP may be superior to APM for *maintenance treatment*, indicating the need for a modification of categorical recommendations discouraging APP in the maintenance treatment of severe psychosis [6]. A systematic review of youth studies spanning 15 years found that APP is also common in children and adolescents, especially with Attention-Deficit Hyperactivity Disorder (ADHD) and oppositional defiant disorder (ODD), with a prevalence of APP among antipsychotic-treated youth of about 10% (6% in child studies and 12% in adolescent studies) [7]. Furthermore, APP has been shown to be used to control aggressive and disruptive behavior in acute pediatric settings, especially in the context of intellectual disability and developmental disorders [8].

Using two or more psychopharmacological medications may be more effective than monotherapy for symptom control, especially if the mechanisms of action are complementary (e.g., antipsychotic-antidepressant or antipsychotic-anticonvulsant combination) [9,10]. Instead, it may be argued that the concomitant use of two or more pharmacologically similar antipsychotics lacks scientific rationale, also exposing the patient to the risk of pharmacokinetic interactions that may have important implications for drug overdosing and adverse neuromotor or metabolic side effects, and thus raising particular concerns for youth populations [11]. Nevertheless, in the real-world clinical experience, APP may sometimes be the last or only feasible option to obtain greater therapeutic response than has been achieved with APM.

We investigated the use of lurasidone as an augmentation treatment of risperidone/aripiprazole in six consecutive cases of children and adolescents with treatment-resistant TS. The eligibility criteria included the presence of symptoms, especially non-tic symptoms such as aggressive behavior and obsessive symptoms, that failed to respond adequately to at least two different antipsychotics and, when deemed appropriate, to respond to a combination with a medication with a different therapeutic indication or chemical class (e.g., antidepressant or anticonvulsant).

## 2. Patient Information

As expected in the context of a neurodevelopmental condition [12,13], some of the TS patients (age range, 11–17 years) presented with additional neurodevelopmental symptomatology (e.g., autistic traits, hyperactivity features or borderline cognitive functioning). However, the severity of the neuropsychiatric symptoms was not such as to fulfill the criteria set by the Diagnostic and Statistical Manual of Mental Disorders, Fifth Edition (DSM-5) for another neurodevelopmental disorder (e.g., autism spectrum disorder, ADHD, or intellectual disability). Nevertheless, the associated psychopathology certainly contributed to making the clinical presentation more complex, with significant implications for treatment response [14], as well as management, overall outcome, and quality of life of patients with such type of TS [15].

According to clinical judgement, reasons for avoiding the administration of medications other than the ones prescribed were the following: (i) suicide risk and/or elevated aggressiveness (e.g., selective serotonin reuptake inhibitors, SSRI) and (ii) poor compliance to treatments requiring strict monitoring (e.g., clozapine or lithium). As an option, APP was extensively discussed with patients’ families in terms of potential benefits and risks, and it was agreed to proceed in the context of individual trials including close monitoring of clinical response, adverse effects, and physical health by medical examinations and blood tests. Notably, all of the patients were also receiving psychological treatment during the observation period, even though we could not formally assess the effect of such an intervention.

Table 1 reports medical and family history of the patients as well as any relevant past pharmacological interventions and outcomes. Every effort was made to conduct the most accurate possible medication reconciliation, comprehensively determining the reasons for psychopharmacological treatment prescriptions (e.g., tics, aggressiveness, and obsessive symptoms); however, such process was not always possible due to the lower quality of certain medical records.

## 3. Clinical Findings

After failure to control aggressive behavior or obsessive symptoms with other single or combined agents, such symptomatic manifestations were regulated by the time of the subsequent control visit planned 30 days later, by using lurasidone as an add-on therapy to risperidone or aripiprazole. The following information is noteworthy: (i) Before the add-on therapy with lurasidone, children themselves and their parents considered the additional symptomatic manifestations unacceptable. (ii) In one case only, tics were sub-optimally controlled with the ongoing treatment and lurasidone add-on appeared to be less beneficial in reducing tics, while reducing aggressive behavior and obsessive symptoms. (iii) In the same case, lurasidone also improved depressive symptoms which were present in comorbidity. (iv) In another case, lurasidone was firstly tried as an add-on therapy to haloperidol, which was already in place; however, despite reducing aggressive behavior and obsessive symptoms, the combination was poorly tolerated due to reported sedation. All clinical data, including therapeutic interventions’ dosage and duration, are organized as a timeline in Table 1.

## 4. Follow-Up and Outcomes

Two physicians independently performed the following: (a) the Clinical Global Impression (CGI) in order to assess (i) severity of illness (before lurasidone add-on, 5.83 ± 1.17 (Mean, M ± Standard Deviation, SD); after lurasidone add-on, 3.17 ± 0.75; t = 8.0, *p* < 0.001), (ii) global improvement, and (iii) treatment efficacy [16] (Table 2); (b) the Modified Overt Aggression Scale (MOAS) in order to assess (i) verbal aggression, (ii) aggression against property, (iii) auto-aggression, and (iv) physical aggression (weighted sum before lurasidone add-on, 12.50 ± 6.32; weighted sum after lurasidone add-on, 5.83 ± 2.64; t = 4.1, *p* = 0.009) [17] (Table 3); and (c) the Children’s Yale-Brown Obsessive Compulsive Scale (CY-BOCS Symptom Checklist) in order to assess severity of (i) obsessions and (ii) compulsions (total score before lurasidone add-on, 22.00 ± 2.97; total score after lurasidone add-on, 15.50 ± 1.05; t = 7.1, *p* < 0.001) [18] (Table 4). In the rare instances of discrepant attribution, consensus was reached through discussion with a third senior clinical researcher. Symptom improvement as experienced by children’s parents was also collected from a narrative perspective. Quotes were extracted by medical records or parents’ written communications (Table 5).

At the time of writing, no significant neuromotor or metabolic side effects had been reported in all cases in a follow-up period ranging from a minimum of 4 months to 6 months, supporting the stability of the observed clinical improvement.

## 5. Discussion

Lurasidone has high-affinity binding to D2 (antagonist), 5-HT2A (antagonist), 5-HT7 (antagonist), and 5-HT1A (partial agonist) receptors, and moderate affinity for noradrenergic α2C and α2A receptors, and a weak affinity for 5-HT2C receptors. It has FDA approval for use in pediatric populations, i.e., adolescent-onset schizophrenia (13–17 years) and bipolar depression (10–17 years). Evidence for its efficacy in children and adolescents with autism spectrum disorder is less conclusive, as an overall improvement in the Clinical Global Impression was not accompanied by an improvement in additional efficacy measures including irritability, hyperactivity, stereotypic behavior, inappropriate speech, lethargy/withdrawal, and obsessive-compulsive symptoms [19,20]. The available studies also support its efficacy in reducing negative symptoms, cognitive dysfunction, and depressive symptoms [4]. Furthermore, apart from a higher risk of akathisia, lurasidone has been suggested to contain metabolic and cardiovascular side effects as compared with prior second-generation antipsychotics, while keeping the risk of extrapyramidal symptoms low [4]. Our findings confirm and extend previous evidence, suggesting a good safety profile of lurasidone in the context of APP, and its effectiveness in reducing aggressive behavior and obsessive symptoms, as well as depressive symptoms in TS.

Increasing evidence-based studies are proving the benefit of APP [21]. In line with the very recent revision that a certain proportion of selected patients can benefit from APP without further negative consequences [21], despite very limited and observed outside of a clinical trial setting, evidence from our case series suggests that lurasidone add-on therapy may be well tolerated and effective in the treatment of complex forms of TS whose additional symptoms, namely aggressive behavior and obsessive symptoms, are refractory to APM or the combination of single antipsychotic medications with other classes of psychopharmacological treatments. Of course, longer follow-up evidence will be needed in order to assess long-term tolerability and efficacy of such polypharmacy. In addition, the efficacy of lurasidone monotherapy as compared with placebo in patients with TS remains to be tested. Furthermore, due to its purely clinical nature, the treatment dose was chosen based on patient’s response, thus requiring investigation of maximum exposure and dose levels in clinical trials. Finally, we cannot exclude a role of regression to the mean and placebo effects in accounting for the observed change. Thus, the nature of the present data, as well as the previous very limited and inconclusive evidence on the efficacy of lurasidone in neurodevelopmental conditions, calls for caution in the treatment of TS with lurasidone.

## 6. Conclusions

While still investigational, the preliminary evidence presented here gives reason to hope that lurasidone might be an effective option in TS, warranting further investigation of its potential benefits in neurodevelopmental conditions.

## Figures and Tables

**Table 1:** Flow chart of pharmacological intervention up to lurasidone add-on therapy to risperidone or aripiprazole in six children/adolescents with Tourette syndrome.

Patient	Age	Gender	Age of Onset	Psychiatric Family History	Psychiatric Comorbidity	Medical Comorbidity	Medication Attempted (Max Dose, Exposure Time) [Age at Time of Administration]	Tics	Aggressive Behavior	Obsessive Symptoms
Child 1	16	Male	11	No	Yes (autistic traits)	Yes (diabetes)	Aripiprazole (??)	Optimal	Suboptimal	Suboptimal
							Aripiprazole (up to 10 Mg/D, 11 months) + fluvoxamine (??, 11 months) [11 years, 7 months]	Optimal	Suboptimal	Suboptimal
							Aripiprazole (10 Mg/D, 11 months) [12 years, 6 months]	Optimal	Suboptimal	Suboptimal
							Aripiprazole (10 Mg/D, 3 months) + olanzapine (up to 7.5 Mg/D, 3 months) [13 years, 5 months]	Optimal	Suboptimal	Suboptimal
							Olanzapine (10 Mg/D, 4 months) [13 years, 8 months]	Optimal	Suboptimal	Optimal
							Olanzapine (10 Mg/D, 9 months) + topiramate (50 Mg/D, 9 months) [14 years]	Suboptimal	Suboptimal	Suboptimal
							Olanzapine (10 Mg/D, 12 months) [14 years, 9 months]	Suboptimal	Suboptimal	Suboptimal
							Aripiprazole (up to 15 Mg/D, 12 months) [15 years, 9 months]	Optimal	Suboptimal	Suboptimal
							Aripiprazole (15 Mg/D, 1 month) + lurasidone (18.5 Mg/D, 1 month) [16 years, 9 months]	Optimal	Optimal	Suboptimal
							Aripiprazole (15 Mg/D, ongoing) + lurasidone (37 Mg/D, ongoing)	Optimal	Optimal	Optimal
Child 2	12	Male	7	Yes (Mother line: tics, hyperactivity, learning disability, depression, anxiety; father line: OCD, depression)	Yes (autistic traits, hyperactivity features, borderline cognitive functioning)	Yes (GERD)	Risperidone (up 0.75 Mg/D, 19 months) [6 years, 7 months]	Suboptimal	Optimal	Suboptimal
							Risperidone (1 Mg/D, 7 months) + aripiprazole (up to 6 Mg/D, 7 months) [8 years, 2 months]	Optimal	Suboptimal	Suboptimal
							Risperidone (1 Mg/D, 12 months) + aripiprazole (6 Mg/D, 12 months) + methylphenidate (10 Mg/D, 12 months) [8 years, 9 months]	Suboptimal	Optimal	Suboptimal
							Risperidone (up to 4 Mg/D, 17 months) + aripiprazole (up to 15 Mg/D, 17 months) + methylphenidate (10 Mg/D, 17 months) + IVIG (15 g, 17 months) [9 years, 9 months]	Optimal	Suboptimal	Suboptimal
							Risperidone (4 Mg/D, 4 months) + aripiprazole (15 Mg/D, 4 months) + clomipramine (up to 20 Mg/D, 4 months) + VPA (up to 600 Mg/D, 4 months) + IVIG (15 g, 4 months) [11 years, 2 months]	Optimal	Suboptimal	Suboptimal
							Risperidone (4 Mg/D, 4 months) + aripiprazole (15 Mg/D, 4 months) + fluvoxamine (up to 100 Mg/D, 4 months) + VPA (600 Mg/D, 4 months) + IVIG (15 g, 4 months) [11 years, 6 months]	Optimal	Suboptimal	Suboptimal
							Risperidone (4 Mg/D, 3 months) + aripiprazole (15 Mg/D, 3 months) + levomepromazine (up to 100 Mg/D, 3 months) + fluvoxamine (100 Mg/D, 3 months) + VPA (600 Mg/D, 3 months) + IVIG (15 g, 3 months) [11 years, 10 months]	Optimal	Suboptimal	Suboptimal
							Risperidone (4 Mg/D, ongoing) + lurasidone (up to 74 Mg/D, ongoing) + VPA (to 600 Mg/D, ongoing) + IVIG (15 g, ongoing)	Optimal	Optimal	Optimal
Child 3	11	Male	??	No	Yes (hyperactivity features, oppositional behavior, borderline cognitive functioning)	No	Antipsychotic not better specified (??, ??)	Optimal	Suboptimal	Suboptimal
							Risperidone (up to 5 Mg/D, at least 11 months) [10 years, 6 months]	Optimal	Suboptimal	Suboptimal
							Risperidone (5 Mg/D, 2 months) + lurasidone (up to 74 Mg/D, 2 months) [11 years, 5 months]	Optimal	Suboptimal	Optimal
							Risperidone (5 Mg/D, ongoing) + lurasidone (up to 148 Mg/D, ongoing)	Optimal	Optimal	Optimal
Child 4	17	Male	??	No	No	No	Aripiprazole (??, ??)	Optimal	Suboptimal	Suboptimal
							Aripiprazole (??, ??) + ziprasidone (??, ??)	Optimal	Suboptimal	Suboptimal
							Aripiprazole (up to 8 Mg/D, ??) + quetiapine (up to 200 Mg/D, ??)	Optimal	Suboptimal	Suboptimal
							Aripiprazole (12 Mg/D, 8 months) + haloperidol (1.5 Mg/D, 8 months) [16 years, 9 months]	Optimal	Suboptimal	Suboptimal
							Aripiprazole (up to 24 Mg/D, 1 month) + haloperidol (up to 3 Mg/D, 1 month) + lithium sulphate PR (166 Mg/D, 1 month) [17 years, 5 months]	Optimal	Suboptimal	Suboptimal
							Haloperidol (3 Mg/D, 20 days *) + lurasidone (74 Mg/D, 20 days) + lithium sulphate PR (166 Mg/D, 20 days) [17 years, 6 months]	Optimal	Optimal	Optimal
							Aripiprazole (up to 30 Mg/D, ongoing) + lurasidone (74 Mg/D, ongoing) + lithium sulphate PR (166 Mg/D, ongoing)	Optimal	Optimal	Optimal
Child 5	12	Male	??	Yes (mother: depression, suicide attempt; sister: mood disorder)	Yes (low mood **)	Yes (Diabetes)	Aripiprazole (10 Mg/D, 10 months) [11 years, 1 month]	Suboptimal	Optimal	Suboptimal
							Aripiprazole (10 Mg/D, 2 months) + risperidone (4 Mg/D, 2 months) [11 years, 11 months]	Suboptimal	Optimal	Suboptimal
							Aripiprazole (10 Mg/D, ongoing) + lurasidone (37 Mg/D, ongoing)	Suboptimal	Optimal	Optimal
Child 6	13	Male	9	No	Yes (hyperactivity features, borderline cognitive functioning)	No	Antipsychotic not better specified (??, ??)	Optimal	Suboptimal	Suboptimal
							Risperidone (6 Mg/D, 7 months) [11 years, 7 months]	Optimal	Suboptimal	Suboptimal
							Aripiprazole (7 Mg/D, 7 months) [12 years, 2 months]	Optimal	Suboptimal	Suboptimal
							Aripiprazole (up to 10 Mg/D, 8 months) + mirtazapine (1 Mg/D, 8 months) [12 years, 9 months]	Optimal	Suboptimal	Suboptimal
							Aripiprazole (10 Mg/D, 1 month) + lurasidone (74 Mg/D, 1 month) [13 years, 5 months]	Optimal	Optimal	Suboptimal
							Aripiprazole (10 Mg/D, ongoing) + lurasidone (111 Mg/D, ongoing)	Optimal	Optimal	Optimal

Mg/D, milligrams per Day; OCD, obsessive compulsive disorder; GERD, gastroesophageal reflux disease; IVIG, intravenous immunoglobulin; VPA, valproic acid; ??, unknown; PR, prolonged release; * discontinued due to reported sedation; ** depressive symptoms were improved by lurasidone; Optimal, clinical impression of symptom improvement >50%; Suboptimal, clinical impression of symptom improvement <50%.

**Table 2:** Clinical Global Impression (CGI).

Patient	CGI-S before Lurasidone Add-On	CGI-S after Lurasidone Add-On	CGI-I	CGI-E Treatment Efficacy	CGI-E Side Effects
Child 1	5	3	2	Moderate	Do not interfere with patient’s functioning
Child 2	6	3	2	Marked	Do not interfere with patient’s functioning
Child 3	7	4	2	Marked	None
Child 4	6	4	3	Moderate	Do not interfere with patient’s functioning
Child 5	4	2	2	Moderate	None
Child 6	7	3	1	Marked	None

CGI-S, CGI-Severity of illness; CGI-I, CGI-Global Improvement; CGI-E, CGI-Efficacy Index.

**Table 3:** The Modified Overt Aggression Scale (MOAS).

Patient	Current Treatment	VA	AAP	AA	PA	Weighted Sum
Child 1	Before lurasidone	3	0	2	2	17
	Lurasidone 18.5 Mg/D	2	0	1	1	9
	Lurasidone 37 Mg/D	1	0	1	1	8
Child 2	Before lurasidone	1	2	0	2	13
	Lurasidone 74 Mg/D	0	1	0	1	6
Child 3	Before lurasidone	1	0	2	3	19
	Lurasidone 111 Mg/D	0	0	2	2	14
	Lurasidone 148 Mg/D	0	0	1	1	7
Child 4	Before lurasidone	0	3	0	2	14
	Lurasidone 74 Mg/D	0	2	0	1	8
Child 5	Before lurasidone	1	0	0	0	1
	Lurasidone 37 Mg/D	1	0	0	0	1
Child 6	Before lurasidone	3	0	0	2	11
	Lurasidone 74 Mg/D	1	0	0	2	9
	Lurasidone 111 Mg/D	1	0	0	1	5

VA, verbal aggression; AAP, aggression against property; AA, auto-aggression; PA, physical aggression; Weighted Sum = (VAx1) + (AAPx2) + (AAx3) + (PAx4).

**Table 4:** Children’s Yale–Brown Obsessive Compulsive Scale (CY-BOCS Symptom Checklist).

Patient	Lurasidone Treatment	Obsessions						Compulsions						
		Time Occupied	Interference	Distress	Resistance	Degree of Control	Sub-Total	Time Spent	Interference	Distress	Resistance	Degree of Control	Sub-Total	**Total**
Child 1	Before	Severe	Moderate	Mild	Mild	Little	10	Severe	Severe	Mild	Mild	Little	11	Moderate (21)
	18.5 Mg/D	Moderate	Moderate	Mild	Mild	Moderate	8	Moderate	Moderate	Mild	Mild	Moderate	8	Moderate (16)
	37 Mg/D	Mild	Moderate	Mild	Mild	Moderate	7	Mild	Moderate	Mild	Mild	Moderate	7	Mild (14)
Child 2	Before	Severe	Severe	Mild	Mild	Little	11	Moderate	Moderate	Mild	Mild	Moderate	8	Moderate (19)
	74 Mg/D	Moderate	Moderate	Mild	Mild	Moderate	8	Mild	Moderate	Mild	Mild	Moderate	7	Mild (15)
Child 3	Before	Severe	Severe	Moderate	Moderate	Little	13	Severe	Severe	Severe	Moderate	Little	14	Severe (27)
	111 Mg/D	Moderate	Moderate	Moderate	Moderate	Moderate	10	Moderate	Moderate	Moderate	Moderate	Moderate	10	Moderate (20)
	148 Mg/D	Moderate	Moderate	Mild	Mild	Moderate	8	Moderate	Moderate	Mild	Moderate	Moderate	9	Moderate (17)
Child 4	Before	Moderate	Moderate	Moderate	Mild	Moderate	9	Severe	Severe	Moderate	Mild	Little	12	Moderate (21)
	74 Mg/D	Moderate	Moderate	Mild	Mild	Moderate	8	Moderate	Moderate	Mild	Mild	Moderate	8	Moderate (16)
Child 5	Before	Moderate	Moderate	Moderate	Mild	Little	10	Moderate	Moderate	Moderate	Mild	Little	10	Moderate (20)
	37 Mg/D	Moderate	Moderate	Mild	Mild	Moderate	8	Moderate	Mild	Mild	Mild	Moderate	7	Mild (15)
Child 6	Before	Severe	Severe	Moderate	Mild	Little	12	Severe	Severe	Moderate	Mild	Little	12	Severe (24)
	74 Mg/D	Severe	Moderate	Moderate	Mild	Moderate	10	Moderate	Moderate	Moderate	Mild	Moderate	9	Moderate (19)
	111 Mg/D	Moderate	Moderate	Mild	Mild	Moderate	8	Moderate	Moderate	Mild	Mild	Moderate	8	Moderate (16)

Mg/D, milligrams per day; Time Occupied/Spent, extreme (4), severe (3), moderate (2), mild (1), none (0); Interference, extreme (4), severe (3), moderate (2), mild (1), none (0); Distress, extreme (4), severe (3), moderate (2), mild (1), none (0); Resistance, extreme (4), severe (3), moderate (2), mild (1), none (0); Degree of Control, no (4), little (3), moderate (2), much (1), complete (0); Total CY-BOCS, extreme (32–40), severe (24–31), moderate (16–23), mild (8–15), subclinical (0–7).

**Table 5:** Parents’ quotes.

Patient	Lurasidone Add-on (dose)	Symptom Improved
Child 1	“Since the beginning of the treatment he seems to be more cooperative and less tied to his control issues!” (18.5 Mg/D)	Aggressive behavior; Obsessive symptoms
	“He now shows fewer obsessive thoughts. He also appears to be more friendly with both his peers and adults.” (37 Mg/D)	
Child 2	“He seems quieter at home now. Also, he’s quite less repetitive about his thoughts. He’s doing well.” (74 Mg/D)	Aggressive behavior; Obsessive symptoms
Child 3	“His behavior and compulsions at home have notably improved.” (111 Mg/D)	Obsessive symptoms
	“His behavior has improved at home and he is now also less worried about strangers and less prone to physically attack them.” (148 Mg/D)	Aggressive behavior
Child 4	“Fidgeting and aggression have greatly faded away since the discharge.” (74 Mg/D)	Aggressive behavior; Obsessive symptoms
Child 5	“Low mood and melancholy have softened. He is still very focused on his things, but the situation seems to be under control.” (37 Mg/D)	Obsessive symptoms
Child 6	“No more episodes of aggression have happened since the introduction of Lurasidone.” (74 Mg/D)	Aggressive behavior
	“He seems less absorbed in his thoughts.” (111 Mg/D)	Obsessive symptoms

Mg/D, Milligrams per Day.

## Data Availability

The data reported in this paper are available from the medical history of the patient.
